# A Text-Book of Medical Treatment: Diseases and Symptoms

**Published:** 1900-12

**Authors:** 


**A Text-Book of Medical Treatment: Diseases and Symptoms.** By NESTOR TIRARD, M.D. Pp. x., 692. London: J. & A. Churchill. 1900.

This book is designed to supply "the links between Physiology and Pathology, between Pharmacology and Therapeutics," which are not always readily apparent; the author hopes "that to senior students this volume may form a useful supplement to their text-books on Medicine, and that, as a work of reference, practitioners may find in it much that is helpful and suggestive." It is to the latter class we should look more especially: the average student finds details of treatment in the ordinary text-books of medicine sufficient for his present needs, but later, after he has fathomed these more or less deeply, he is glad to get assistance from such books as this when called upon to do everything possible in a critical and intractable case. At whatever part of the book it may be opened we find a full and emphatic account of what can be done for the disease in question, and the account is not largely encumbered with various suggestions which are obviously useless and often opposed to common sense. Every page is instructive, and the suggestions will commend themselves to the reader as having been founded on experience and good judgment. It is difficult to criticise where all is good, and there must be many points where opinions may differ: for instance, we read (page 37) that "patients with chlorosis generally require to be encouraged to take outdoor exercise," and no mention is made of the necessity for absolute rest in bed of many patients who cannot make good blood so long as they continue to get about more or less actively. Again (page 50), we are told that in acute coryza, quinine, prescribed in doses of one or two grains three times a day, "will lower the temperature." How often has this fallacy been repeated! We are expressly told that the drug "must be used with some moderation, since, if given in over-doses, it may produce headache and buzzing in the ears." Surely it has long ago and repeatedly been proved that doses which will not produce these symptoms will also fail to have any antipyretic effect; in fact, that quinine as an antipyretic has long ago been replaced by drugs which are more efficient as antipyretics and which bring no corresponding discomforts with them. Again (page 44), we should prefer a stronger statement as to the question of operation in exophthalmic goitre, for we are told that "operative measures . . . have occasionally been recommended, but they have not hitherto been generally adopted." This scarcely summarises the results of present experience, which tends to show that however satisfactory have been the results of operation in ordinary goitre, the risks of operation in exophthalmic goitre are too great to be undertaken.

We venture to predict a popularity for the book which will render subsequent editions necessary at no long intervals.

